# Glymphatic and lymphatic communication with systemic responses during physiological and pathological conditions in the central nervous system

**DOI:** 10.1038/s42003-024-05911-5

**Published:** 2024-02-24

**Authors:** Ester Licastro, Giuseppe Pignataro, Jeffrey J. Iliff, Yanxiao Xiang, Eng H. Lo, Kazuhide Hayakawa, Elga Esposito

**Affiliations:** 1 Neuroprotection Research Laboratories, Departments of Radiology and Neurology, Massachusetts General Hospital, Harvard Medical School, Charlestown, MA USA; 2 Division of Pharmacology, Department of Neuroscience, School of Medicine, University “Federico II”, Naples, Italy; 3 Department of Anesthesiology and Perioperative Medicine, Oregon Health & Science University, Portland, OR USA; 4 Department of Pharmacy, Qilu Hospital of Shandong University, Jinan, Shandong China; 5 Consortium International pour la Recherche Circadienne sur l’AVC (CIRCA), Radcliffe Department of Medicine, University of Oxford, Headington, Oxford UK

**Keywords:** Neuroscience, Inflammation

## Abstract

Crosstalk between central nervous system (CNS) and systemic responses is important in many pathological conditions, including stroke, neurodegeneration, schizophrenia, epilepsy, etc. Accumulating evidence suggest that signals for central-systemic crosstalk may utilize glymphatic and lymphatic pathways. The glymphatic system is functionally connected to the meningeal lymphatic system, and together these pathways may be involved in the distribution of soluble proteins and clearance of metabolites and waste products from the CNS. Lymphatic vessels in the dura and meninges transport cerebrospinal fluid, in part collected from the glymphatic system, to the cervical lymph nodes, where solutes coming from the brain (i.e., VEGFC, oligomeric α-syn, β-amyloid) might activate a systemic inflammatory response. There is also an element of time since the immune system is strongly regulated by circadian rhythms, and both glymphatic and lymphatic dynamics have been shown to change during the day and night. Understanding the mechanisms regulating the brain-cervical lymph node (CLN) signaling and how it might be affected by diurnal or circadian rhythms is fundamental to find specific targets and timing for therapeutic interventions.

## Abstract (web summary)

Glymphatic and lymphatic communication contribute to the clearance of cerebrospinal fluid and the removal of macromolecules from the brain. Understanding the intricate mechanisms and the circadian influence might revolutionize treatment strategies.

## Introduction

The glymphatic system was first explained^1^ as a macroscopic waste clearance system that utilizes astroglial (glial-lymphatic) channels to eliminate soluble proteins and metabolites from the central nervous system. Since then, an updated model for clearance of brain interstitial solutes now includes four segments of brain fluid transport: (1) periarterial CSF influx, (2) interstitial solute movement, (3) efflux along the perivenous spaces (that contributes to the already known cranial and spinal nerves CSF efflux) and (4) meningeal lymphatic drainage^2^.

Tissue homeostasis and efficient elimination of waste products - such as protein aggregates or toxic metabolites (e.g., amyloid-b) were traditionally attributed to intracellular and extracellular mechanisms. This includes cellular and protein degradation and efflux of solutes to the bloodstream through the blood-brain barrier (BBB)^3^. After the report of Iliff et al. ^1^, more evidence has shown how this clearance system might also help to distribute non-waste molecules such as lipids^4^, glucose^5^, nutrients and neurotransmitters within the brain^6^. Further studies suggested that the glymphatic activity might have daily rhythm and that the clearance of toxic compounds, attributed to the glymphatic system, works mostly during sleep^6^.

Although physically detached, the glymphatic and meningeal lymphatic systems seem to be functionally connected. Animal studies have demonstrated that cerebrospinal fluid (CSF) drains via meningeal lymphatic vessels into cervical lymph nodes (CLN)^7^ and further human studies have shown similar connections^8,9^. CSF flow into cervical lymph nodes (CLN) can regulate the trafficking of immune cells^10^. Inflammatory mediators can induce lymphangiogenesis^11^ by VEGFC-VEGFR3 binding^12^. This signaling has been showed to also regulate the inflammatory process in pathological conditions like focal cerebral ischemia^13^.

Ischemic stroke, occurring when blood supply to part of the brain is interrupted or reduced, is a leading cause of disability and death for which no acute treatments exist beyond recanalization. However, despite the decreasing numbers in stroke risk and post-stroke disability, many neuroprotectants have failed in clinical stroke trials, and new therapies for both acute and chronic stroke are still needed^14^. Time of stroke onset might also need to be taken into consideration^15,16^. In the context of stroke, there may be a circadian pattern, e.g., strokes in patients occur mostly in the morning (8 AM) and in the evening (8 PM), as the second peak^16^.

Circadian biology modulates all aspects of mammalian physiology and disease. Circadian “circa diem” rhythms are daily cycles of physical and behavioral changes regulated by a highly phylogenetically conserved system. Circadian clocks are found in all cells of CNS and peripheral organs. The central clock located in the suprachiasmatic nuclei (SCN) of the brain synchronizes other internal clocks through chemical and physical cues, light-based signals, and non-light-based signals. Meanwhile, factors like body temperature, hormone levels, and patterns of eating and fasting^17^ could impact the peripheral circadian clocks regulated by the SCN.

The circadian system regulates a variety of critical cellular processes, including aspects of inflammation^18,19^, metabolism^20^ and cell redox homeostasis^21^.

During day-time/light phase, humans are awake and in their active phase, opposite to night-time/dark phase when they are asleep and inactive. However, most of the preclinical studies are done in rodents, nocturnal animals, where the phases are opposite to humans. In rodents, day-time/light phase corresponds to their asleep/inactive phase, while night-time/dark phase corresponds to their asleep/active phase. It was hypothesized that diurnal or daily rhythms may affect stroke mechanisms and neuroprotection in rodent models of cerebral ischemia^15^. Because rodents are nocturnal, preclinical stroke studies that are performed in the daytime correspond to their inactive or sleep phase. In contrast, clinical trials are mostly performed during human active or awake phase. Moreover, another study showed that there are significant diurnal effects on the immune response after focal cerebral ischemia in mice^22^. Therefore, understanding how circadian rhythm affects stroke would help define targets, finding biomarkers and potential therapy in stroke.

In this mini-review, we will introduce the CNS clearance system with particular focus on the meningeal lymphatic uptake and drainage into cervical lymph nodes and how this pathway might be important in pathological events, such as stroke. Some attention will be given to the daily effect on waste clearance as well as in CNS pathologies.

## Glymphatic system in the regulation of cerebrospinal fluid transport

Cerebrospinal fluid (CSF) is a clear, colorless plasma-like fluid that bathes the CNS. CSF circulates through a system of cavities: ventricles, subarachnoid space in the brain and the central canal of the spinal cord^23^. The CSF is mainly secreted by the choroid plexus epithelium, which is located within the lateral, third and fourth ventricles and flows through the four ventricles into the subarachnoid space of the cortex and spinal cord. The estimated secretion of CSF is around 150-270 milliliters within the CNS^24^. It has been suggested that CSF production in humans may be subjected to circadian regulation with a peak in CSF production during the night^25,26^, however, more studies are needed.

The glymphatic system is a glial-dependent waste clearance pathway in the central nervous system of vertebrates. This system supports the perivascular exchange of CSF and interstitial solutes throughout the brain. According to this model from the subarachnoid compartments, the CSF is transported into perivascular spaces (PVS)^27^ through areas called Virchow-Robin space, i.e., penetrating arteries that surround the brain parenchyma^28,29^.

The CSF influx through the interstitium is facilitated by aquaporin-4 (AQP4)^1^, a water channel expressed in the astrocyte end-feet that communicates with the interstitial fluid (ISF), aiding in the removal of toxic compounds through the PVS. Ultimately, the efflux fluids are drained into lymph vessels existing in the meninges and transported out of the CNS to the bloodstream through cervical lymphatic vessels^30,31^.

It is still unclear what drives CSF flows into the PVS. Modeling has helped to investigate several mechanisms^32^. It seems that the forces are relatively small (i.e., peristalsis created by intravascular blood pressure pulses^33^) and may originate from several mechanisms^34^, including arterial pulsation^35,36^, cardiac systolic pressure^36,37^, respiration^38^, CSF pressure gradients and sleep^6,39^. CSF clearance pathways might also be altered by intracranial pressure elevation^40^.

However, additional research is necessary to determine the precise proportions of physiological contributions to modeling CSF influx. Tangible biological discoveries and modeling are both crucial for a comprehensive understanding of this system.

Age is also an influencing factor of the glymphatic system, whose activity is reduced during the aging process^41–43^. These factors drive bulk flow of CSF, facilitating glymphatic ISF–CSF exchange and clearance function^35,44^.

As demonstrated in rodents by Iliff et al. ^35^ the administration of Dobutamine, an adrenergic agonist, enhances cardiac contractility and arterial pulsatility, resulting in higher CSF penetration in brain parenchyma. These findings were supported by other works where mice, subjected to internal carotid artery ligation, showed the opposite effects^45^. Taken together, these studies might suggest that cardiac failure could potentially reduce glymphatic fluid exchange, through the reduction of vascular tone. However, more studies are needed to validate this hypothesis.

Intriguingly, as stated above, it has been demonstrated that glymphatic system is predominantly active during sleep. Natural sleep, observed in rodents, has been associated with enhanced periarterial CSF influx and improved waste material clearance, including soluble amyloid-β (Aβ)^6^. It is now clear that during sleep, interstitial space volume is increased^6^, and this in turn could be a consequence of reduced locus coeruleus–dependent noradrenergic activity. However, no direct correlation between locus coeruleus activity and glymphatic system has been shown.

In humans, similar findings have been replicated wherein sleep was able to enhance glymphatic clearance efficiency compared to awake states^46^ and a night of sleep deprivation^47^. Overall, these findings may suggest that the restorative function of sleep may shift brain into a functional state that facilitates the glymphatic clearance of waste products of neural activity accumulating during wakefulness^48^.

## CSF transport and clearance under circadian rhythm

Circadian rhythms are defined as biological rhythms with a period of ~24 h. In order to be classified as circadian, a biological rhythm endogenously generated from a self-sustained oscillator can be synchronized to an environmental cycle (i.e, by the light/dark cycles) and temperature compensated^49,50^. Circadian rhythms are driven by circadian clocks found in all cells of CNS and peripheral organs. The master clock in the suprachiasmatic nuclei (SCN) of the brain regulates other central clocks via chemical and physical stimuli, photic signals, and non-photic signals. Meanwhile, body temperature, hormone metabolites, and feeding/fasting cycles may influence SCN-regulating peripheral circadian clocks^51^. The circadian system regulates a variety of critical cellular processes, including inflammation^18,19^, metabolism^20^ and cell redox homeostasis^21^. These cellular mechanisms are altered in many pathologies including stroke.

Some evidence has revealed how glymphatic function changes during the day, with a peak of activity at mid-day, when mice are in their inactive state (mostly asleep)^52^. Furthermore, it has been demonstrated that differences in glymphatic influx, solute clearance, and CSF drainage to the lymph nodes are regulated by circadian rhythms^52^. Specifically, it has been showed that in the awake mice CSF distribution is dependent on two main factors: (1) periarterial influx suppressed during brain active state, and (2) reduced expression of AQP4 polarized that in turn prevents CSF/ISF exchange by reducing the interstitial space volume^3,6^. Indeed, circadian glymphatic function is sustained by circadian regulation of AQP4 polarization in astrocytes, whose genetic deletion produces an absence of day/night differences in CSF distribution and drainage to the lymph node^52^. Astrocytes, expressed also in the suprachiasmatic nucleus (SCN), are actively involved in sustaining circadian oscillation^53,54^, and regulate bulk fluid movement through CSF/ISF exchange across the brain under circadian control^52^.

Sleep, a state of immobility characterized by reduced responsiveness and rapid reversibility, is an extremely complicated process. Over the past years, there have been many attempts to identify a purpose for why we sleep, few theories have been proposed, including the restorative theory^55^. Based on this theory, we sleep to allow the body to reaper biological processes altered during the awake time^56^. Despite decades of efforts, the mechanisms underlying the restorative function of sleep and how its disruption or circadian disruption (alteration of the daily circadian rhythms^57^) impairs brain functions is only partly unraveled. The primary goal remains to comprehend how these processes impact glymphatic function and lymphatic drainage, aiming to prevent the associated comorbidities linked to sleep misalignment (out of phase with the light/dark cycle^58^).

## Molecular targets of the glymphatic system in the treatment of stroke

Looking ahead, focusing on the mechanism driving the glymphatic system’s role in the brain could be pivotal in enhancing neurological function recovery and enhancing patient outcomes post-stroke. AQP4 may represent a target for therapeutic purpose with potential application on stroke therapy^59–61^. AQP4 might indeed be implicated in the edema spreading after stroke, where CSF could be partly responsible for edema formation^62^.

Although several potential AQP modulators have been developed^63,64^, clinic trials have failed due to related pharmacokinetic issues - lack of selectivity, stability and toxic side effects.

Some studies have identified microRNAs (miRNAs), small non-coding RNAs that regulate post-transcriptional gene expression^65^; moreover, it has been shown their involvement as endogenous modulators of AQP expression^66^. This identification has opened new perspectives for therapeutic interventions and provides an alternative approach to target these proteins.

Few findings highlighted how circulating miRNA patterns are also implicated in the induction of ischemic tolerance^67,68^, i.e., ischemic preconditioning and postconditioning. Ischemic tolerance is defined as an endogenous neuroprotective phenomenon, induced by a small ischemic event, able to protect an organ from a subsequent/previous lethal ischemic event^68,69^. These adaptive processes became attractive, allowing the prospective implementation of personalized therapies^70^.

Interestingly, several miRNAs have been correlated in cerebral ischemia. For instance, miR-320a was reported to inhibit AQP1 and AQP4 gene expression both in vitro and in vivo in a cerebral ischemia rat model, whereas anti-miR-320a upregulated AQP1 and AQP4 expression with consequent reduction of infarct volume^71^. Other studies, instead, showed how AQP4 down-regulation mediated by miR-145^72^, miR-130b^73^ and miR-29b^74^ can exert a protecting role against ischemic stroke. Specifically, AQP4 silencing, associated with an increase of miR-224 and miR-19a expression, could be responsible for decreased astrocyte connectivity and fluid movement in the cerebral parenchyma^75^. However, it is still under debate and needs further investigation how the reduced protein expression shown in stroke models might, in turn, influence AQP4 localization on the astrocytic end-feet and, finally, the glymphatic flow.

It has been demonstrated that Sur1-Trpm4 (a non-selective cation channel) and AQP4 are able to form a complex “chansporter” involved in the worsening of ischemic damage due to astrocytic swelling^76,77^. Probably, the interaction of ion channels and solute transporter may involve other protein channels, such as the Na + -K + -Cl− cotransporter (NKCC1), the acid-sensing ion channel 1a (ASIC1a), Na + /H+ exchanger isoform 1 (NHE1), Na + /Ca2+ exchanger or K+ channels, crucial factors in the dysregulation of ion homeostasis in the CNS under ischemic conditions^78–81^. During CNS injury, these proteins can result dysregulated and their hyperactivation generates an excessive influx of cations (sodium and calcium) worsening brain damage due to ischemic reperfusion. An open question remains: how are these systems involved in the fluid movement in the glymphatic system? The answer may highlight a novel therapeutic target in cerebral ischemic stroke.

## The meningeal lymphatic system-mediated CSF clearance

The lymphatic system mediates the drainage of interstitial fluid (ISF, bodily fluid naturally produced via trans-capillary blood exchange which surrounds cells and tissues) and regulates immune cell trafficking and surveillance in most mammalian tissues^10^.

Evidence of the existence of a lymphatic-like system involved in cerebrospinal fluid (CSF) drainage to peripheral LNs goes back to the middle of the 20th century^82–84^. Yet, it wasn’t until 2015, thanks to the advancement of more intricate techniques capable of identifying detailed structural and functional traits, that two separate studies unequivocally demonstrated the existence of a lymphatic vessel network within the mouse brain’s dura mater and its link to the cervical lymph nodes^7,85^.

Compared to peripheral lymphatics, meningeal lymphatics are composed of a less ramified network of thin-walled initial lymphatic vessels^86^. Brain meninges are constituted by three layers: dura, arachnoid, and pia mater. Meningeal lymphatic vessels are situated in the external meningeal layer, the dura.

Lymphatic vessels drain components of the cerebrospinal fluid (CSF) that fills the subarachnoid space. CNS-draining into the lymphatics has been recognized as important step for CNS homeostasis. Initial studies proposed that meningeal lymphatic vessels in the dorsal part of the skull, were mainly involved in the clearance of cerebrospinal fluid (CSF). Subsequently, the significance of meningeal lymphatic vessels positioned in the lower part of the skull has been recognized, emphasizing their anatomical placement and structural characteristics that aid in the absorption and flow of cerebrospinal fluid (CSF)^87^.

The glymphatic and meningeal lymphatic systems, though physically distinct, are functionally linked. Studies by three models using pharmacological, surgical, and genetic approach demonstrated that impaired meningeal lymphatic function alters the flow of CSF macromolecules through the paravascular route. For instance, Da Mesquita et al. found that surgically ligating meningeal lymphatics reduced the accumulation of a tracer in cervical lymph nodes injected into the cisterna magna^88^. This ligation also led to decreased CSF influx and ISF efflux into the brain tissue. Moreover, this pathway is affected by aging^89^, as older mice display reduced brain perfusion by CSF macromolecules compared to younger counterparts^41^. This decline in brain perfusion appears linked to deteriorating lymphatic vasculature, potentially influencing various age-related pathologies.

## The meningeal lymphatic-mediated immune regulation

The meningeal lymphatic vessels might also be involved in the maintenance of some meningeal immune cells. Indeed, different types of immune cells can be found in the meninges and meningeal spaces, specifically in the arachnoid space. In the absence of inflammation or infection, these cells are retained within the meningeal spaces. Impairment of lymphatic vessels, either systemically or locally, results in accumulation of T lymphocytes in the meninges^90^, suggesting that these vessels are somehow involved in maintaining the homeostatic immune cell number. In single-cell transcriptomic studies that have explored the immune cells populating the meningeal compartment, multiple immune cell types, including B cells^91,92^, macrophages^93^ but mostly T lymphocytes (CD4), have been shown to be important for the brain function^94^. Therefore, immune cells present in the meninges and meningeal spaces, and their homeostatic regulation by lymphatic vessels, might be important for the maintenance of brain function.

## The brain to cervical lymph nodes connection via CSF drainage

The idea that the CNS is a privileged, isolated compartment from the rest of the body has largely been replaced by the concept that crosstalk between CNS and systemic biology is also important in health and disease^95,96^. Historically, the central nervous system has been considered lacking in lymphatic vasculature. Lymphatic circulations were thought to only extend throughout most of the body and contribute to tissue homeostasis but not in the brain^97^. However, today, many studies have showed the presence of a lymphatic vessel network in the dura meninges and the CSF transport into deep cervical lymph nodes through lymphatic vessels^7,82–85^.

Cervical lymph nodes are located in the neck region. Indeed, they are also known as the lymph nodes of the neck. Their traditional function is to filter and transport lymph from surrounding lymph nodes and viscera back into the bloodstream. In pathological conditions, they are involved in protecting the body against infection by delivering immune cells, lymphocytes, to areas where the immune response has been triggered^87,98^.

Few animal studies have demonstrated that cerebrospinal fluid (CSF) drains via meningeal lymphatic vessels into cervical lymph nodes (CLN)^7,85,88,89^ and some human studies have also shown similar connections^9,99^. Because CSF transports fluid from the brain into deep cervical lymph nodes through lymphatic vessels, this pathway might also be responsible for clearing the brain from harmful metabolites. Interestingly, an increase in CSF movement and waste clearance have been associated with sleep^52^. Indeed, while the glymphatic function is increased during sleep, the drainage of CSF to the lymph nodes is higher during awake time. After cisterna magna injection, in vivo imaging of mandibular lymph nodes showed increased tracer outflow during the night compared with day, when entry of CSF into the brain is low. This day/night difference in lymph node filling persists in constant conditions and is absent in animals without AQP4, suggesting that circadian rhythm mechanisms are responsible for these differences^52^. However, more studies are needed to investigate how daily rhythm affects the brain-CLN signaling.

## Pathological relevance of brain to CLN connection

The lymphatic vessels (LVs) are composed of lymphatic endothelial cells (LECs)^100,101^. Studies available so far show that meningeal lymphatic cells are originated from endothelial cells in a process VEGFR3 dependent. Vascular endothelial growth factor (VEGF)-C has been shown to stimulate lymphangiogenesis by binding its receptor, VEGFR-3^12^. Lymphangiogenesis is a dynamic process during embryogenesis but in the adult, it only takes place during certain pathological conditions such as inflammation, tissue repair, and tumor growth^11^.

Pathological conditions might influence brain-CLN signaling by lymphangiogenesis. How damaged brains initially send signals to trigger systemic inflammation is still an open question. The underlying pathway involved in pathological conditions might be through brain-to-cervical lymph node signaling. After focal cerebral ischemia the cerebrospinal fluid drains into cervical lymph nodes and the pathway induces lymphangiogenesis along with upregulations of oxidative stress and inflammatory cytokines^13^.

Rat and mouse models of focal cerebral ischemia along with co-cultures of lymphatic endothelium and macrophages, demonstrated the activation of lymphatic endothelium in cervical lymph nodes (CLNs) following ischemic stroke via VEGF-C/VEGFR3 signaling. Additionally, inhibiting VEGFR3 signaling pharmacologically or surgically removing superficial CLNs mitigated post-stroke inflammation and decreased brain damage. Specifically, lymphatic endothelial cells isolated from CLNs at 3 h after reperfusion followed by microarray analysis demonstrated that the transcriptome was rapidly altered. Gene Set Enrichment Analysis (GSEA) suggested that differentially expressed genes were largely related to matrix pathways and transmembrane receptor protein tyrosine kinase activity. One of the most upregulated genes included CCL28, known to regulate lymphatic endothelial migration. The blockade of VEGFR3 tyrosine kinase with MAZ51 treatment significantly reduced CCL28 in superficial CLNs after cerebral ischemia. Altogether, these data might suggest that brain-to-CLN signaling is responsible for triggering systemic inflammatory responses after acute stroke^13^. However, another study using a mouse model of the active experimental autoimmune encephalomyelitis (EAE) shows that VEGFR3 blockade in mice induced dural lymphatic vessel impairment and was insufficient to block autoimmune neuroinflammation^102^, implicating that lymphatic inflammation can have complex double-edged sword actions of either one can worsen acute tissue damage, or under some conditions the other mechanism can also help resolve damage and promote repair^103,104^.

**Fig. 1:**
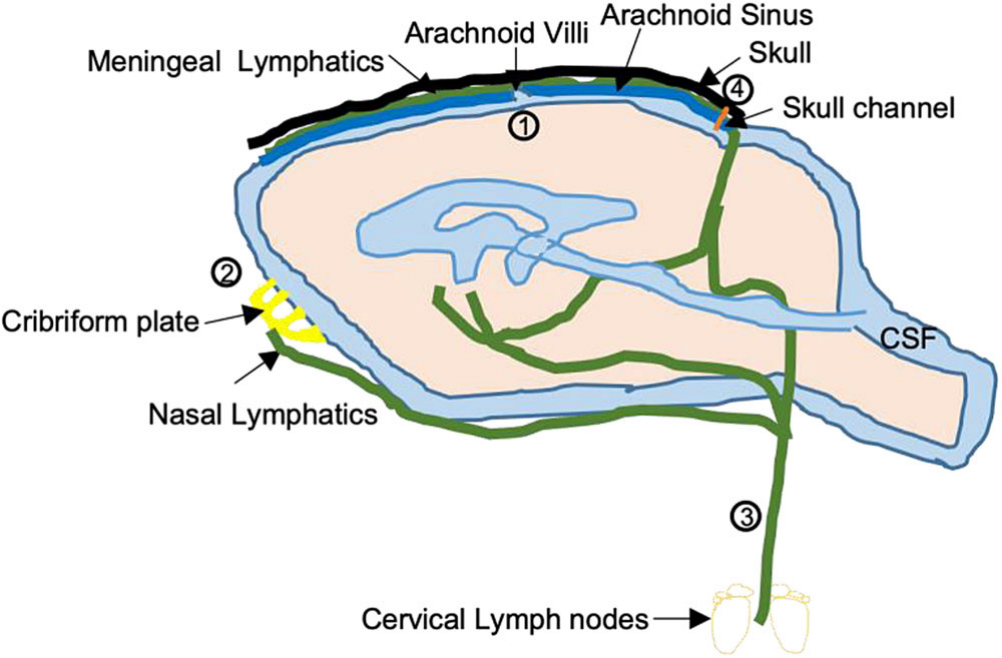
CSF outflow pathways. **1** CSF flows through arachnoid villi, found along the superior sagittal venous sinus, into the blood **2** It drains through the cribriform plate in association with the olfactory nerves. From this location, CSF is absorbed into nasal mucosal lymphatics. It does eventually reach the CLNs **3** It flows from the meningeal lymphatics directly to cervical lymph nodes **4** CSF transits into the skull marrow trough skull channels.

How lymphatic drainage can refresh CSF in animals and humans is still controversial. In animal models, CSF outflow seems to occur via several routes: through arachnoidal villi^105^, along spinal and cranial nerves^106^, along dural vessels that transit skull channels into the marrow^107^ in addition to dural lymphatics to cervical lymph nodes pathway^89^. Studies also show evidence for CSF outflows through arachnoid villi, perineural routes, and dural lymphatics in humans. However, it has been showed that even if CSF flows in the parasagittal dura, the dura mater adjacent to the superior sagittal sinus with high density of lymphatic vessels^108^, this route does not seem to be the major efflux for CSF^109^ (Fig. 1).

Several MRI studies in humans have suggested the existence of brain lymphatic networks linked to cervical lymph nodes, mirroring the observations made in mice, indicating potential clinical relevance^8,9^. Furthermore, emerging data from experimental models and clinical trials may now support the feasibility of directly injecting therapeutics into LN to block inflammation^110,111^.

There is now evidence that brain-lymphatic-CLN signaling might be involved in other CNS pathologies such as stroke, multiple sclerosis (MS)^90,112^, aging, Alzheimer’s disease (AD)^88,113^ and Parkinson’s disease (PD)^114^. In stroke animal models, as stated before, brain to CLN pathway has been suggested to be involved in the system inflammatory response. Moreover, in stroke patients, neuronal glutamate receptor antigens and myelin basic protein fragments have been detected in CLNs^115^, suggesting communication between brain and CLNs. MS is an immune-mediated inflammatory disorder that results in progressive damage to the human CNS. Evidence that cervical lymph nodes are involved in B and T cell mediated immunological reactions, in the CNS, has been shown in many experimental studies. In particular, the ablation of meningeal lymphatics diminishes pathology and reduces the inflammatory response of brain-reactive T cells in an animal model of multiple sclerosis, linking the brain-CLN signaling to the pathophysiology of MS^90,112^. For Parkinson’s disease, a study in PD mouse model, showed how meningeal lymphatics, draining oligomeric α-syn into the lymph nodes, might contribute to macrophage activation and to the peripheral inflammation^114^. In Alzheimer’s disease, a few studies suggested that the lymphatic system might represent an important step for the clearance of β-amyloid. Plus, Amyloid-beta has been found in human lymph nodes^88,113^.

**Fig. 2:**
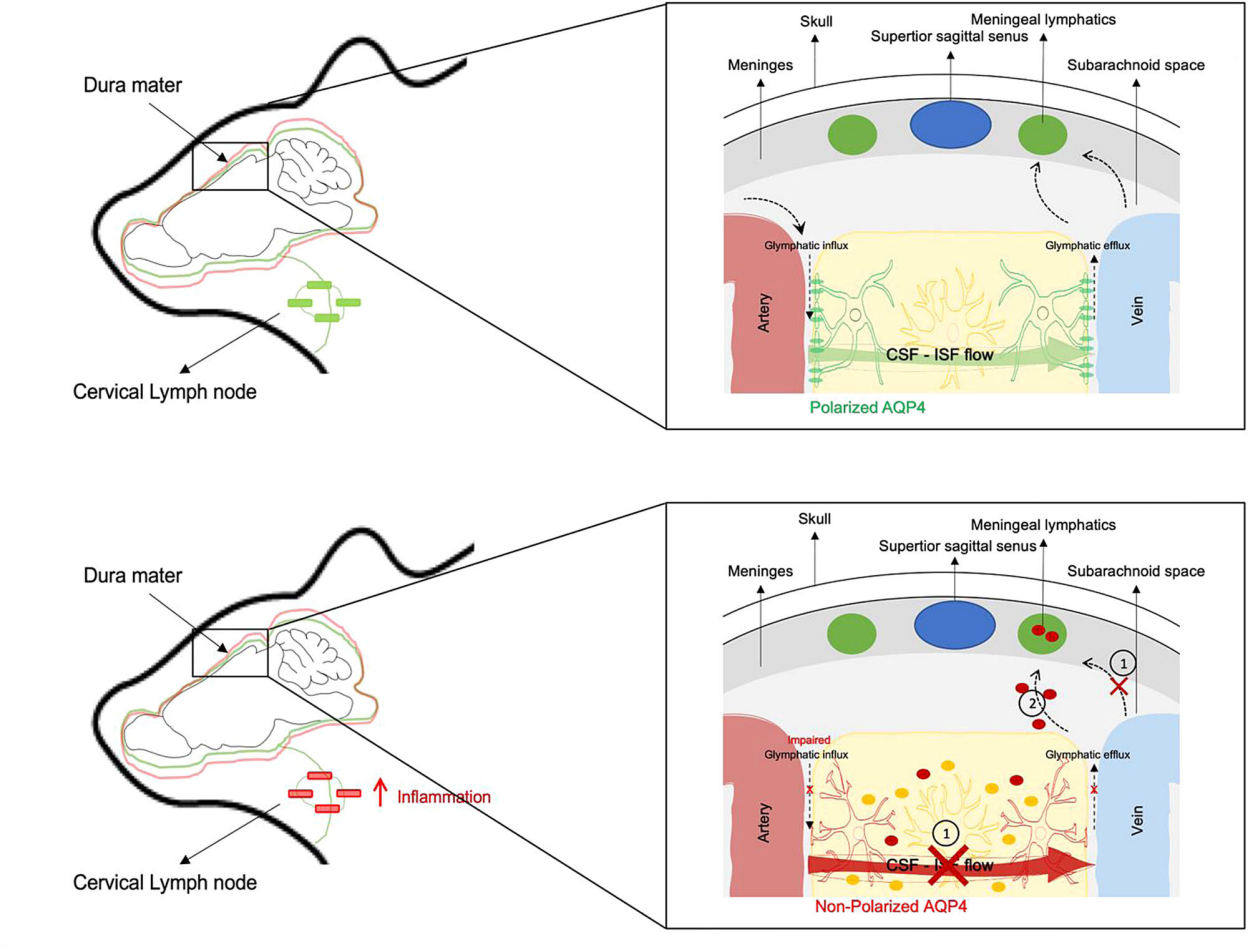
Glymphatic system and meningeal lymphatic system. The glymphatic system drains CSF into the brain via a periarterial pathway, while interstitial fluid (ISF) leaves the brain through the perivenous pathway. CSF, containing macromolecules and immune cells, can flow from the brain parenchyma through the dura meningeal lymphatics into the lymph nodes and extracranial systemic circulation. In some pathological conditions **1** Altered expression of polarized AQP4 prevents CSF/ISF exchange by reducing the interstitial space volume, reducing the waste clearance **2** The meningeal lymphatic vessels transport CSF, containing solutes coming from the brain (such as VEGFC, oligomeric α-syn, β-amyloid) into the cervical lymph nodes, activating inflammatory response.

All these studies, in different CNS pathologies, underline how important it is to understand the mechanisms regulating the brain-CLN pathway (Fig. 2).

## Conclusion

The intricate interplay between the glymphatic and meningeal lymphatic systems holds pivotal implications for brain health and disease. These systems, although physically distinct, collaboratively contribute to the clearance of cerebrospinal fluid (CSF) and the removal of macromolecules from the brain. While their dysfunction has been implicated in various central nervous system (CNS) pathologies, such as stroke^13,62^ and other neurological disorders^9,38,116–120^, the precise molecular mechanisms underlying these dysfunctions remain elusive.

Emerging insights suggest potential fluctuations in brain clearance mechanisms throughout the day, potentially being more active during sleep. Understanding the intricate mechanisms governing glymphatic/lymphatic interactions and their connection to cervical lymph nodes, along with the influence of circadian biology on these pathways, represents a promising frontier for further exploration.

Unraveling the intricate mechanisms governing glymphatic/lymphatic dynamics, especially their communication with the lymph nodes, could give us insights into pathological conditions affecting the CNS. Moreover, understanding the mechanisms of circadian influence on these pathways might revolutionize treatment strategies by pinpointing optimal intervention timings.

Future investigations should, therefore, steer toward unraveling the molecular complexities of glymphatic to meningeal lymphatic-mediated clearance pathways as well as the response of drained CLNs, delving deeper into their malfunctioning in diverse CNS pathologies. Moreover, understanding the precise influence of circadian rhythms on CSF production and clearance mechanisms warrants rigorous exploration to unveil potential therapeutic targets. This multifaceted understanding could potentially spearhead the development of precision therapies, strategically timed interventions, and innovative treatment modalities tailored to harness the natural ebb and flow of functioning brain mechanisms, thereby reshaping the landscape of neurological disorder management.

### Reporting summary

Further information on research design is available in the Nature Portfolio Reporting Summary linked to this article.

### Supplementary information

Reporting Summary
